# Application of Traditional Japanese Drug Jidabokuippo in a Modern Society

**DOI:** 10.3389/fphar.2022.853012

**Published:** 2022-05-23

**Authors:** Hajime Nakae, Yasuhito Irie, Toshiharu Kitamura, Manabu Okuyama

**Affiliations:** Department of Emergency and Critical Care Medicine, Akita University Graduate School of Medicine, Akita, Japan

**Keywords:** trauma, inflammatory swelling, made-in-Japan, static blood, adverse event

## Abstract

**Background:** Jidabokuippo (JDI) (治打撲一方) has been used in Japan to alleviate contusion-induced swelling and pain since medieval times.

**Method:** This review investigated the effects of JDI on various symptoms in patients with trauma or static blood^[TM1]^. The PubMed and Igaku Chuo Zasshi databases were searched until 24 December 2021. We summarize the benefits of applying JDI to inflammatory conditions, including bruises.

**Results:** JDI has been used to resolve blood ^[TM1]^ stasis, regulate qi in trauma patients, and treat inflammatory swelling and pain caused by rheumatoid arthritis and cellulitis. As the adverse event rate associated with JDI is low (1.3%), JDI is considered a safe drug.

**Conclusion:** JDI can be used to resolve blood^[TM1]^ stasis in trauma patients without adverse events associated with nonsteroidal anti-inflammatory drugs.

## Introduction

**FIGURE 1:**
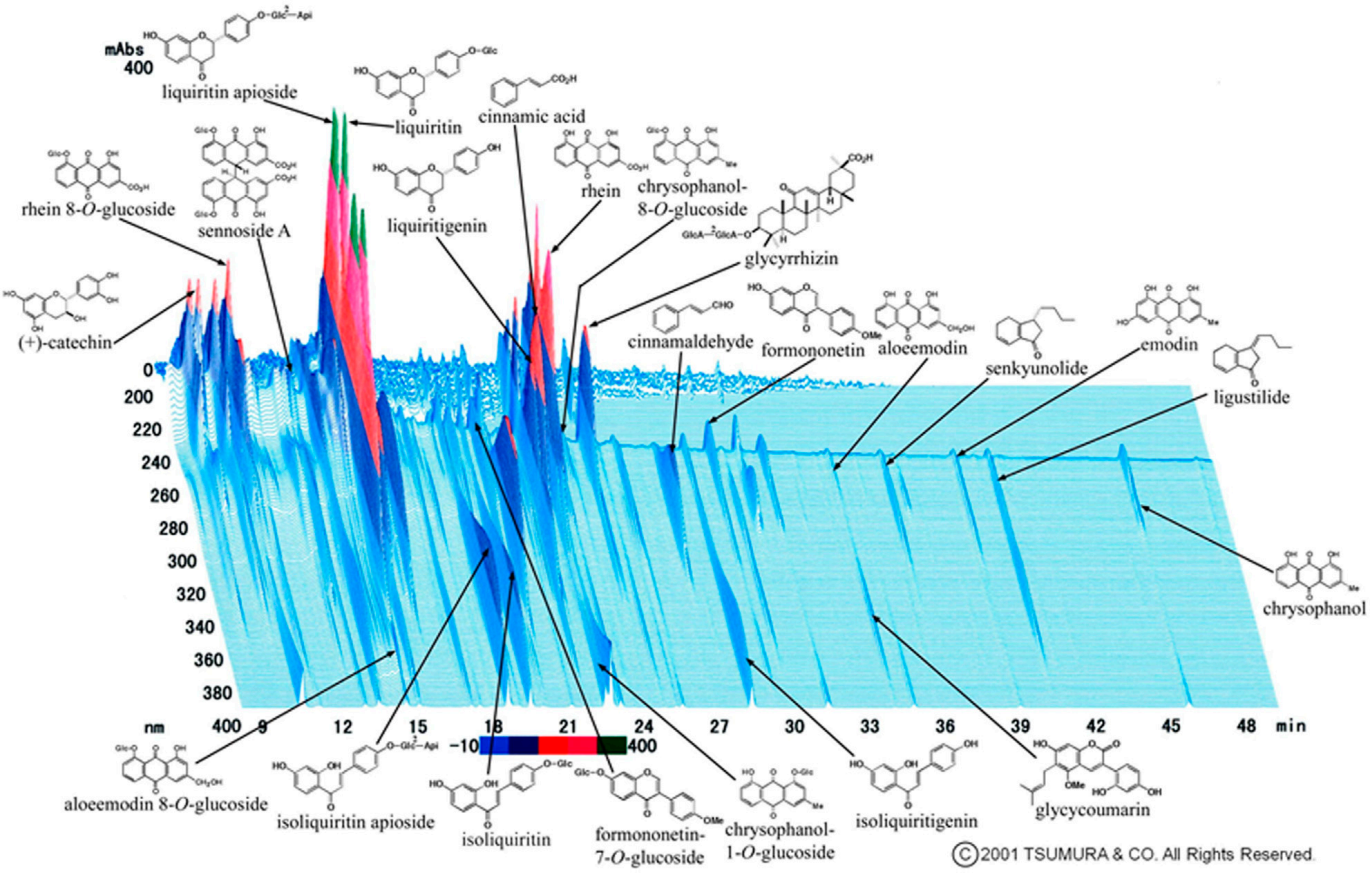
Three-dimensional high-performance liquid chromatography profile of jidabokuippo. *Nuphar japonica* DC. contains major ingredients: nupharidine, deoxynupharidine, nupharamine, and nupharin. *Quercus acutissima* Carruth. contains quercitrin, scopoline, fraxin, and tannic acid. *Ligusticum officinale* (Makino) Kitag. contains cnidilide, neocnidilide, ligustilide*, senkyunolide*, butylphthalide, butylidenephthalide, pregnenolone, vanillin, coniferyl ferulate, ferulic acid, and scopoletin. *Neolitsea cassia* (L.) Kosterm. contains cinnamaldehyde*, cinnamyl acetate, phenylpropyl acetate, cinnamic acid*, and salicylaldehyde. *Syzygium aromaticum* (L.) Merr. and L.M.Perry contains acetyleugenol, chavicol, caryophyllene, humulene, caryophylla, eugenocide, eugeniin, higenamine, rhamnetin, and kaempferol. *Rheum palmatum* L. contains sennoside A*–F, rhein*, aloe emodin*, emodin*, chrysophanol*, naphthalene, catechin*, epicatechin, and cinnamic acid*. *Glycyrrhiza glabra* L. contains glycyrrhizin*, glabric acid, liquiritin*, liquiritin apioside*, liquiritigenin*, isoliquiritin*, licoricidin, licoricone, licoflavone, formononetin*, glycerol, and glycycoumarin*.*Shown in Panel 1).

Jidabokuippo (JDI) (治打撲一方) is an herbal mixture used in Japan to alleviate contusion-induced swelling and pain. It is composed of *Nuphar japonica* DC., *Quercus acutissima* Carruth., *Ligusticum officinale* (Makino) Kitag., *Neolitsea cassia* (L.) Kosterm., *Syzygium aromaticum* (L.) Merr. and L.M.Perry, *Rheum palmatum* L., and *Glycyrrhiza glabra* L. (Table 1; Figure 1) (Department of Pharmacognosy and DPPN, 2018; Sakakibara, 2008; Nakae and Irie, 2020).

**TABLE 1:** Pharmacological action of formulated crude drugs in jidabokuippo.

Crude drug	Composition ratio (g)	Efficacy in Kampo medicine	Pharmacological action
*Nuphar japonica* DC.	3.0	Resolving blood^[TM1]^ stasis and stomachic property	Analgesia, diuresis, and anti-edematous action
*Quercus acutissima* Carruth	3.0	Resolving blood^[TM1]^ stasis and antidiarrheal action	Boosting and convergence
*Ligusticum officinale* (Makino) Kitag	3.0	Resolving blood^[TM1]^ stasis, regulating qi, removing wind^[TM1]^ and dampness^[TM1]^, and pain-relieving	Central inhibition, telangiectasia, antithrombotic action, spasmolytic, increasing action on digestive tract mucosa blood flow volume, the elevation of skin temperature, and immunostimulation
*Neolitsea cassia* (L.) Kosterm	3.0	Releasing exterior, descending qi, resolving blood^[TM1]^ stasis, pain-relieving	Perspiration and antipyretic, sedation and spasmolytic, telangiectasia, decreasing blood pressure, antithrombotic action, anti-inflammation, antibacterial action, antitumor action, and regulation of water metabolism
*Syzygium aromaticum* (L.) Merr. and L.M.Perry	1.0	Warming spleen^[TM1]^, stomach^[TM1]^, and kidney^[TM1]^, and descending qi counterflow	Anti-inflammatory, antibacterial action, antiviral action, sedation, and spasmolytic
*Rheum palmatum* L	1.0	Purgative, heat^[TM1]^-clearing, and resolving blood^[TM1]^ stasis	Catharsis, antibacterial action, psychotropic, anti-inflammatory, immunostimulation, lipid metabolism, and antithrombotic action
*Glycyrrhiza glabra* L	1.5	Descending qi, sedative action, relaxing tensions, pain-relieving, warming, and tonifying action, relieving purgative action, preserving fluid^[TM1]^, and stomachic property	Sedation and spasmolytic, antitussive action, anti-inflammatory, antitumor action, antibacterial action, and antiviral action

TM1: traditional medicine module 1

Herein, we document that JDI treatments have been applied to bruises and various inflammation conditions since medieval Japanese society. Potentially relevant articles were identified through a PubMed and Igaku Chuo Zasshi (ICHUSHI) literature search using the keywords (jidabokuppo OR jidabokuippou) for articles published until 24 December 2021. ICHUSHI contains bibliographic citations and abstracts from more than 2,500 biomedical journals and other serial publications published in Japan. Since Kampo medicine targets many intractable and rare diseases and the course of treatment differs in each case, it is difficult to conduct large-scale randomized controlled trials and secure high-quality evidence. Therefore, a case report and case series are also included.

## Source

Kampo prescriptions developed by Japanese expert clinicians in the Edo era were called “honchokeikenho” and are thought to include JDI. In the Sengoku era, the age of provincial wars (1467–1615), some traumatologists called “kinsoi” used drugs that resembled JDI for sword wounds. Shuan Kagawa, who lived from 1683 to 1755, finalized JDI and collected information on treating bruises. It was originally named “ippo (一方)” for “bruise” in “Ippondo-iji-setsuyaku.” Sohaku Asada, a well-known Kampo medicine expert who practiced during the late 19th century (between the end of the Edo era and the early Meiji era), was the first to call it JDI in “Futsugo-yakushitsu-hokan-kuketsu” published in 1878. He reported that Shuan Kagawa developed JDI (Asada, 1981; Morikubo, 1999; Nakae and Irie, 2020).

## Application of Jidabokuippo in the Classical Period

Shuan Kagawa reported that *Quercus* bark has the potential to resolve blood^[TM1]^ stasis and improve fluid congestion found in bruises in “Ippondoyakusen (Ippondoyakusen, 2021).” Contusion and pain caused by trauma are considered static blood^[TM1]^, a sign of a microcirculatory disorder, and JDI alleviates blood^[TM1]^ stasis patterns (Morikubo, 1999). Gentatsu Matsuoka, who lived from 1668 to 1746, reported that *Nuphar japonica* should be used for bruises. Prescriptions that included it were especially effective for bruises in “Yoyakusuchi (Yoyakusuchi, 2021).” Sohaku Asada explained that JDI improved myalgia and ostealgia caused by trauma; *Nuphar japonica* improved blood flow, and *Quercus acutissima* alleviated ostealgia. These two crude elements were the principal agents. *Aconitum carmichaelii* Debeaux with warm meridian is added in the chronic stage in “Futsugo-yakushitsu-hokan-kuketsu” (Asada, 1981). He also explained that dokoppito (土骨皮湯), composed of *Quercus acutissima*, *Carthamus tinctorius* L., *Glycyrrhiza glabra*, *Bupleurum falcatum* L., and *Curcuma zedoaria* (Christm.) Roscoe improved eczema capitis and ostealgia. In dokoppi, also known as Bokusoku, *Quercus acutissima* has strong potential of releasing exterior in “Futsugo-yakushitsu-hokan-kuketsu.”

*Rheum palmatum* has sedative effects in addition to resolving blood^[TM1]^ stasis (Sumida et al., 1988). *Ligusticum officinale*, *Neolitsea cassia*, and *Syzygium aromaticum* have the potential to regulate qi (Table 1). Wada Tokaku, who lived from 1742 to 1803, stated “It is not good resolving blood^[TM1]^ stasis using *Carthamus tinctorius* and *Biancaea sappan* (L.) Tod. for bruises. The regulating qi method should be chosen for this purpose. Provide sedation using shigyakusan (四逆散) or jinkokokito (沈香降気湯)” (Shosozatsuwa, 2021). Since both resolving blood^[TM1]^ stasis and regulating qi should be performed for the treatment of bruises, JDI is thought to have the ideal composition of crude drugs.

Kampo formulations are made from several crude drugs, with each crude drug having several constituents. Therefore, Kampo prescriptions are considered interaction-based multicomponent medicines. The blending effect of crude drugs in JDI is shown in Table 2.

**TABLE 2:** Blending effect of formulated crude drugs in jidabokuippo.

Blended crude drug	Efficacy in Kampo medicine
*Nuphar japonica* + *Ligusticum officinale*	Reducing fluid congestion in a bruise and relieving pain
*Quercus acutissima* + *Ligusticum officinale*	Resolving blood^[TM1]^ stasis, healing bruise, wound, and hematoma
*Quercus acutissima* + *Nuphar japonica*	Improving blood circulation and relieving pain in blood^[TM1]^ stasis
*Quercus acutissima* + *Rheum palmatum*	Clearing heat^[TM1]^ and resolving blood^[TM1]^ stasis
*Syzygium aromaticum* + *Neolitsea cassia*	Warming and improving blood circulation and healing congestive disease
*Neolitsea cassia* + *Glycyrrhiza glabra*	Descending qi counterflow and tranquilization

TM1: traditional medicine module 1

## Application of Jidabokuippo in the Modern Period

The relevance of JDI in modern society is the same as that in its classical use. In short, swelling caused by trauma is diagnosed as blood^[TM1]^ stasis, and JDI is applied to resolve it (Table 3) (Yamamoto, 1975; Hijikata et al., 2007; Futenma et al., 2014; Irie and Nakae, 2019; Yoshinaga et al., 2020).

**TABLE 3:** Previous reports of more than 10 cases using jidabokuippo.

No.	References	Study design	Injuries and diseases	Number of cases
1	Ikeda et al. (1986)	Case series	Trauma	109
2	Kita et al. (1995)	Case series	Rheumatoid arthritis	12
3	Takagi (1995)	Cohort study	Chronic pain caused by trauma	18
4	Sudo and Oribe (2005)	Cohort study	Chronic pain caused by trauma	23
5	Sudo (2005)	Cross-sectional study	Spinal compression fracture	24
6	Sakurai et al. (2006)	Case series	Facial injuries	13
7	Takeda (2010)	Randomized controlled study	Anterior tibiofibular ligament injury	17
8	Nakae et al. (2012)	Randomized controlled study	Rib fractures	76
9	Minamitani (2014)	Case series	Fractures and severe contusions	10
10	Nakae et al. (2015b)	Case series	Fractures of extremities	50
11	Yoshida (2015)	Case series	Facial contusions	47
12	Nakae et al. (2016)	Case series	Trauma	643
13	Hasegawa et al. (2016)	Cohort study	Trauma/postoperative swelling	53
14	Suzuki and Yoshida (2016)	Cross-sectional study	Postoperative finger swelling	112
15	Saito et al. (2019)	Case series	Obstetrics and gynecology patients	112
16	Akiyama et al. (2020)	Case series	Head injury	18
17	Kitamura et al. (2022)	Cross-sectional study	Trauma/postoperation	1,104

Yamamoto reported that JDI was effective for bruises in acute and chronic settings. JDI was much more effective than keishibukuryogan (桂枝茯苓丸), and aconite tuber should be added to JDI in the chronic stage. He also recommended treatment-induced diarrhea using JDI and *Rheum palmatum* in acute severely injured patients, regardless of stool consistency (Yamamoto, 1975).

Plants contain various antioxidants that protect organisms from injury caused by ultraviolet radiation. Kampo formulations have antioxidant and multiple bioactive properties (Table 1) (Nakae, 2011; Hirayama et al., 2018). Yamane evaluated the radical scavenging potentials of seven herbs [*Rheum palmatum*, *Uncaria gambir* (W.Hunter) Roxb., *Syzygium aromaticum*, *Paeonia lactiflora* Pallas, *Glycyrrhiza glabra*, *Polyporus umbellatus* Fries, and *Prunus persica* (L.) Batsch] and reported that the scavenging potential of diphenylpicrylhydrazyl (DPPH) was the highest in *Rheum palmatum*, followed by *Syzygium aromaticum* (Yamane et al., 2000). Tani suggested that polyphenol is closely involved in antioxidant effects based on a positive correlation between the polyphenol content and DPPH radical scavenging potential of herbs (Tani et al., 2004). They investigated 25 herbs. The polyphenol content was highest in *Rheum palmatum*, followed by *Quercus acutissima*, *Nuphar japonica*, *Glycyrrhiza glabra*, *Syzygium aromaticum*, and *Neolitsea cassia*. The DPPH radical scavenging potential was high in *Rheum palmatum*, followed by *Quercus acutissima*, *Nuphar japonica*, *Syzygium aromaticum*, and *Neolitsea cassia*. In addition, *Ligusticum officinale* have anti-inflammatory and antioxidant effects. A study designed to evaluate the effect of herbal extracts in suppressing reactive oxygen formation in human neutrophils showed a suppressive action by *Ligusticum officinale* (Luo et al., 1993). In addition, this herb protects organisms from radiation-induced damage (Ohta et al., 1987; Shinoda, 1995) and protects against edema (Tahara et al., 1998). *Neolitsea cassia* suppresses the formation of reactive oxygen in aqueous extracts (Toda et al., 1991), inhibits O_2_ formation in macrophages (Imamichi et al., 1990), and protects against radiation disorders (Ohta et al., 1987). *Rheum palmatum*, containing anthraquinones, suppresses lipid peroxide formation in human neutrophils (Mian et al., 1987), and condensed tannins have radical scavenging activity (Uchida et al., 1988). *Glycyrrhiza glabra* has anti-inflammatory and edema-suppressing activities (Kumagai, 1982; Amagaya et al., 1984). In addition, *Glycyrrhiza glabra* protects organisms from radiation (Ohta et al., 1987). Thus, JDI includes herbs with antioxidant effects; these herbs may act synergistically to exert antioxidant effects.

We have previously demonstrated the antioxidant activity of JDI in a clinical setting (Nakae, 2010a). Swelling related to trauma occurs due to the enhanced permeability caused by the overproduction of chemical mediators such as free radicals. JDI may improve the pathological condition through these antioxidant properties.

In the clinical setting, Kampo prescriptions should be first administered in doses two to three times greater than the common starting doses in patients with severe symptoms (Nakae and Irie, 2020; Nakae et al., 2021).

**FIGURE 2:**
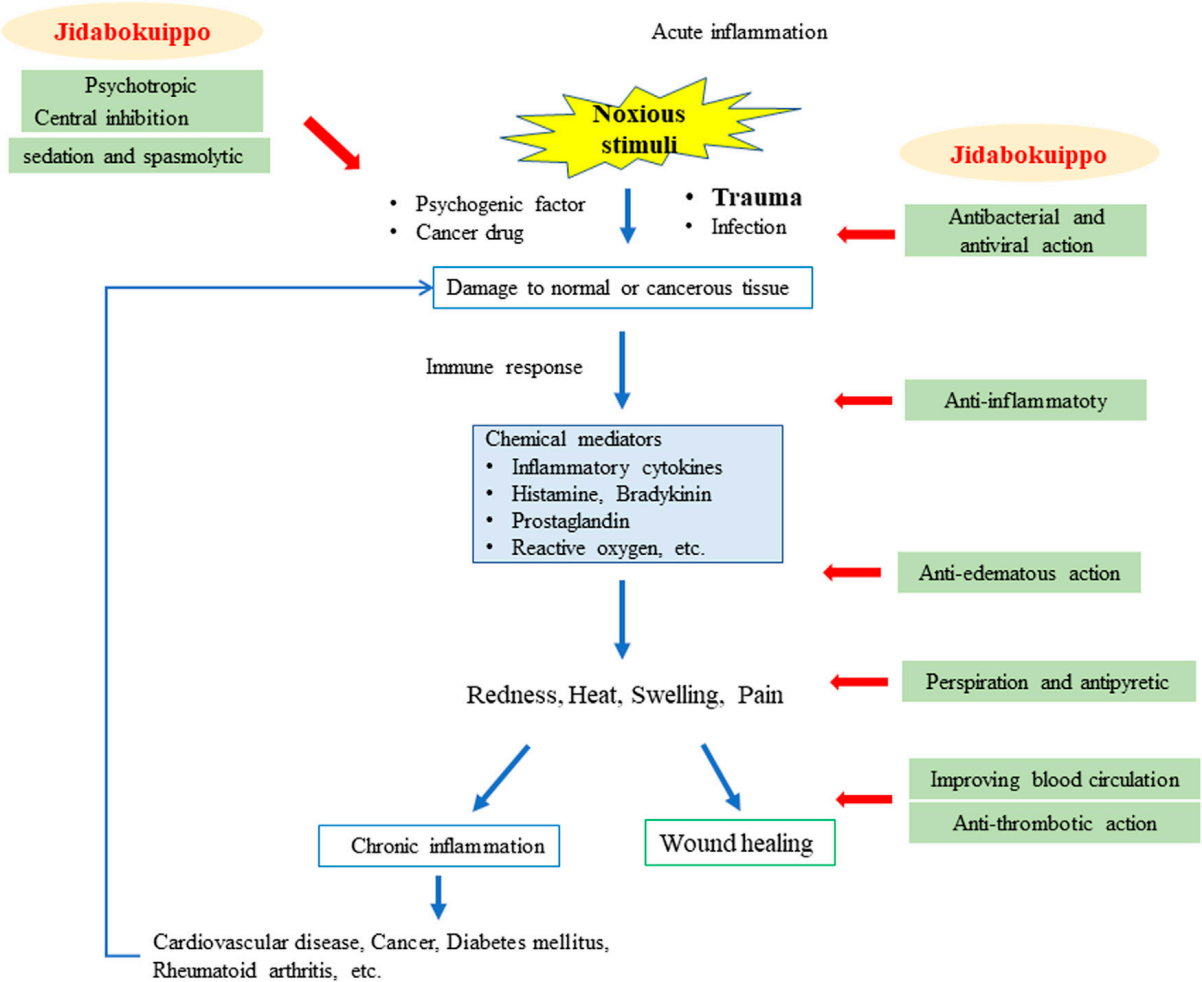
Hypothetical mechanisms of jidabokuippo for acute and chronic inflammation.

The hypothetical mechanisms of JDI are shown in Figure 2. A patient’s signs and symptoms are diagnosed based on theories of Kampo medicine such as yin and yang, deficiency and excess, cold^[TM1]^ and heat^[TM1]^, exterior^[TM1]^ and interior^[TM1]^, six-stage patterns, qi, blood^[TM1]^, fluid^[TM1]^, and zang-fu organs. The patient is to be treated based on those patterns. When a patient’s pattern is in static blood^[TM1]^ and qi depression, JDI is applied to the pattern, regardless the patient’s condition being acute or chronic inflammation.

## Effectiveness of Jidabokuippo

As for the effectiveness of JDI as compared with Western drugs, there are only two randomized controlled studies (Table 3). Takeda compared the efficacy of JDI and nonsteroidal anti-inflammatory drugs (NSAIDs), loxoprofen, in patients with anterior tibiofibular ligament injuries by analyzing the treatment duration using a visual analog scale and girth ( Takeda, 2010). The results showed that compared to loxoprofen, JDI could shorten the swelling duration 2 weeks after the administration. We compared the efficacy of JDI and NSAIDs in patients with rib fractures by analyzing the treatment duration. Our results suggest that compared to NSAIDs, JDI could shorten the treatment duration and may be a promising analgesic agent for both medical and economic reasons (Nakae et al., 2012.).

We have used JDI for various trauma such as rib fractures, fractures of extremities, abdominal wall hematoma, and traumatic asphyxia (Nakae et al., 2012; Nakae et al., 2015a; Nakae et al., 2016; Kitamura et al., 2022; Nakae et al., 2015b; Nakae et al., 2020.).

Suzuki reported that the JDI group had a significantly more robust remission effect than the non-JDI group in postoperative finger swelling (Suzuki and Yoshida, 2016). Nagashima reported that a 35-year-old man with massive subcutaneous swelling after decompressive craniectomy for head trauma showed a rapid reduction of swelling after JDI administration (Nagashima et al., 2018). Furthermore, JDI was applied for chronic subdural hematoma, puncture hematoma after angiography, and subgaleal hematoma with skull fractures (Tsugane et al., 2011; Yoshida et al., 2018; Nakao and Kaneko, 2019; Yamada et al., 2019). The use of JDI has also been applied to treat rheumatoid arthritis and cellulitis (Kita et al., 1995; Nogami et al., 2003; Yoshinaga et al., 2021). Since JDI has antioxidant activity and also inhibits prostaglandin production, its indications may be broader. In Kampo medicine, peripheral neuropathy is often diagnosed as related to blood^[TM1]^ deficiency, static blood^[TM1]^, fluid^[TM1]^ retention, and kidney^[TM1]^ deficiency (Shimada, 2005). Therefore, JDI can resolve blood^[TM1]^ stasis in these indications (Uemura et al., 2013; Narai et al., 2014; Okamoto, 2015; Yabe et al., 2018). Furthermore, JDI has been used for unexplained perineal pain and wasp stings (Nakae, 2013a; Ogata et al., 2019).

Concomitant use of NSAIDs or other Kampo medicines may be necessary for multiple injuries should severe inflammatory reactions occur and severe pain persist (Table 4) (Yonemitsu, 2017; Iwata, 2020). *Aconitum carmichaelii* should be added to JDI when swelling and pain persist. In Kampo medicine, *Aconitum carmichaelii* can move old blood^[TM1]^ stasis (Yamamoto, 1975; Takamura et al., 2018). We previously reported that the *Aconitum carmichaelii* had analgesic and hyperthermic activity and increased blood flow (Nakae, 2008; Nakae et al., 2008; Nakae, 2010b; Nakae, 2010c; Nakae, 2010d; Nakae, 2013b; Nakae et al., 2014). Drug treatment with carbamazepine and pregabalin, nerve block injection, acupuncture, and moxibustion treatment combined with JDI were administered to treat neuropathic pain (Kase et al., 2009; Imaizumi et al., 2016; Suzuki et al., 2017; Okuno and Gi, 2019; Yano et al., 2020).

**TABLE 4:** Concomitant use of Kampo medicine with jidabokuippo.

Kampo medicine	The main potential in Kampo	Knock-on effect of using
Keishibukuryogan	Resolving blood^[TM1]^ stasis	No constipation
桂枝茯苓丸		
Tsudosan	Resolving blood^[TM1]^ stasis	Constipation
通導散		
Sokeikakketsuto	Regulating fluid^[TM1]^	Chronic situation, numbness
疎経活血湯		
Goreisan	Regulating fluid^[TM1]^	Head injury, whiplash injury (headache, nausea)^a^
五苓散		
Eppikajutsuto	Regulating fluid^[TM1]^	Severe cellulitis
越婢加朮湯		

TM1: traditional medicine module 1

a Goreisan (五苓散) should be administered before jidabokuippo (治打撲一方) use.

## A Possible Choice for Jidabokuippo

**FIGURE 3:**
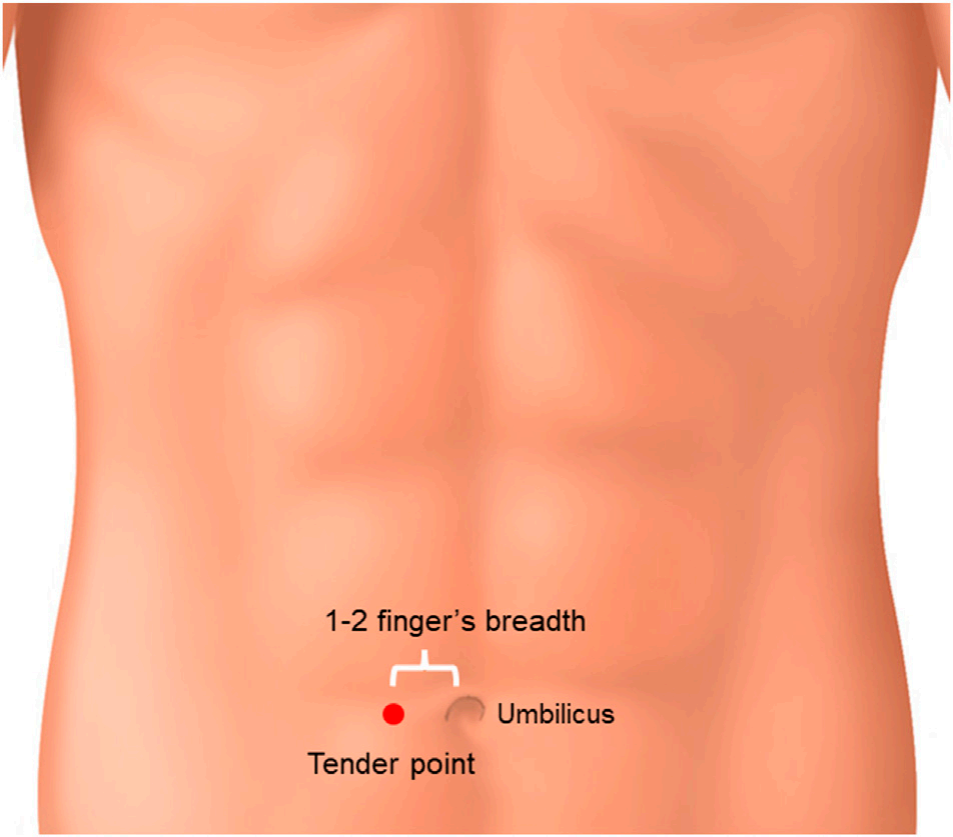
Tender point of jidabokuippo. When abdominal tenderness at the right side of the paraumbilical site was observed, JDI might have been effective.

Takagi reported that JDI was effective in patients with abdominal tenderness at the right side of the paraumbilical site before treatment (Figure 3) (Takagi, 1995). This tender point is considered to indicate blood^[TM1]^ stasis (Morikubo, 1999; Sudo and Oribe, 2005; Suzuki et al., 2017). It is difficult to confirm whether Takagi’s suggestion could be used in the absence of this tender point.

## Safety of Jidabokuippo

The incidence of adverse events associated with Kampo formulations remains unclear. Kitamura et al. studied the adverse events in 1,104 patients who had JDI prescribed (Kitamura et al., 2022). The reported adverse event rate was 1.3%, falling within a low rate of previous reports (0–6.4%) (Ikeda et al., 1986; Kita et al., 1995; Takagi, 1995; Sudo and Oribe, 2005; Sudo, 2005; Sakurai et al., 2006; Takeda, 2010; Nakae et al., 2012; Minamitani, 2014; Nakae et al., 2015a; Yoshida, 2015; Nakae et al., 2016; Hasegawa et al., 2016; Suzuki and Yoshida, 2016; Saito et al., 2019; Akiyama et al., 2020). The most common adverse event was digestive symptoms (0.9%), with diarrhea caused by *Rheum palmatum* being the most common. The adverse event rate of glycyrrhiza-induced pseudoaldosteronism was 0.33% (Table 5). The adverse event rate associated with JDI use is low, and the onset is relatively rapid. Kon reported that the laxative action that accompanies decreased aquaporin-3 expression due to sennoside A in *Rheum palmatum* was mitigated by the anti-inflammatory effects of glycyrrhizin (Kon et al., 2018). Glycyrrhizin is considered to attenuate the adverse events caused by sennoside A. However, we need to pay attention to the pharmacological action of *Rheum palmatum* and *Glycyrrhiza glabra* before concluding that JDI is a safe drug.

**TABLE 5:** Adverse events related to jidabokuippo.

Adverse event		Frequency^a^	Causative crude drugs
Digestive symptom	Diarrhea, loose stool, and abdominal pain	0.47% (10/2,138)	*Rheum palmatum*
	Nausea and vomiting	0.28% (6/2,138)	Combination of crude drugs
	Stomach heaviness	0.09% (2/2,138)	Combination of crude drugs
	Loss of appetite	0.05% (1/2,138)	Combination of crude drugs
Pseudoaldosteronism	Weight increase, edema, hypokalemia, and feeling of weakness	0.33% (7/2,138)	*Glycyrrhiza glabra*
Skin symptom	Rash	0.09% (2/2,138)	*Neolitsea cassia*

a Calculated according to the previous reports (Sudo and Oribe, 2005; Sakurai et al., 2006; Takeda, 2010; Nakae et al., 2012; Nakae et al., 2015a; Yoshida, 2015; Hasegawa et al., 2016; Nakae et al., 2016; Saito et al., 2019; Kitamura et al., 2022).

## Conclusion

NSAIDs are often used to treat pain associated with trauma. However, NSAIDs intake often induces gastrointestinal symptoms. In addition, the use of selective cyclooxygenase-2 inhibitors poses a risk of ischemic heart disease (Hippisley-Cox and Coupland, 2005), and physicians hesitate to use them in patients with a history of cardiovascular disease. In recent years, proton pump inhibitors (PPIs) have been used to prevent NSAID-induced ulcers. However, PPIs pertain to medical economics, fractures, community-acquired pneumonia, watery stools, etc. (Bombardier et al., 2000; Dalton et al., 2009). JDI can be used as an alternative drug under such conditions. Moreover, JDI may be applied to non-trauma patients with blood^[TM1]^ stasis. A large randomized controlled trial is necessary to establish JDI treatment for various diseases with blood^[TM1]^ stasis.
